# 879. Burns Injuries During a Large-scale Conflict: Epidemiology, Severity, and Outcomes from a National Trauma Registry

**DOI:** 10.1093/jbcr/irag033.365

**Published:** 2026-04-06

**Authors:** Josef Haik, Mor Rittblat, Dmitry Kotovich, Shiran Katabi, Adam J Rose

**Affiliations:** Sheba Medical Center, Ramat Gan, HaMerkaz; Hebrew University of Jerusalem Faculty of Medicine, Jerusalem, Yerushalayim; Hebrew University of Jerusalem Faculty of Medicine, Jerusalem, Yerushalayim; Hebrew University of Jerusalem Faculty of Medicine, Jerusalem, Yerushalayim; Hebrew University of Jerusalem School of Public Health, Jerusalem, Yerushalayim

## Abstract

**Introduction:**

High-intensity conflicts precipitate spikes in burn burden that challenge evacuation, triage, and definitive burn care. In particular, blast-related thermal exposure and confined environments heighten the risk of facial burns and inhalation injury, escalating needs for rapid airway control and critical care. We analyzed injury patterns, treatment, and outcomes during the Israel’s 2023–2025 war period to guide surge planning and clinical practice.

**Methods:**

Retrospective registry study of conflict-related casualties from October 27, 2023 to January 19, 2025. We included patients with burns, with or without other trauma, and abstracted demographics, mechanism of injury, anatomic distribution, transport mode, early interventions, total body surface area (TBSA), ICU admission (Y/N), time from event to hospital, and time from event to the OR, disposition, and length of stay. Descriptive analyses were conducted for the overall cohort and for the subset who were hospitalized.

**Results:**

Among 4972 casualties, 479 sustained burn injuries (median age 22; 99.6% male). The predominant mechanism was explosions (85%). Almost half of the injured (47%) were air-evacuated. Common regions were the face (45%), upper extremities (38%), lower extremities (19%), neck (11%), and head (8.6%). In the subset of burn patients who were hospitalized (n = 235), median event-to-hospital interval was 68 minutes (IQR 55–93). Documented inhalation injury was present in 19%. Upon ED arrival, 37% proceeded directly to the OR, and 50% of all casualties were ultimately admitted to the ICU. Early prehospital and ED resuscitation included whole blood in 17% and freeze-dried plasma in 8.8%. Median hospital length of stay was 10 days (IQR 3–20). Among those hospitalized, 49% were discharged to rehabilitation.

**Conclusions:**

In this conflict, burns were overwhelmingly explosion-related and frequently involved the face and upper extremities, with one in two hospitalized patients requiring ICU care. Rapid evacuation (median ≈1 hour) coincided with sizable early use of blood products, yet the inhalation injury burden underscores the need for early airway assessment and standardized ventilatory pathways.

**Applicability of Research to Practice:**

Findings support (1) plastic-surgery–led triage prioritizing facial/hand burns and suspected inhalation injury for expedited airway protection and bronchoscopy; (2) combine TBSA category with airway dependence and need for intervention in OR to anticipate ICU demand.

**Funding for the study:**

This work was supported by the Milgrom Family Support Program for Projects in Military Medicine.

